# INA-RESPOND: a multi-centre clinical research network in Indonesia

**DOI:** 10.1186/s12961-015-0024-9

**Published:** 2015-07-29

**Authors:** Muhammad Karyana, Herman Kosasih, Gina Samaan, Emiliana Tjitra, Abu Tholib Aman, Bachti Alisjahbana, Fatmawati, M. Hussein Gasem, Mansyur Arif, Pratiwi Sudarmono, Suharto, Tuti P. Merati, Clifford Lane, Siswanto, Sophia Siddiqui

**Affiliations:** Center for Applied Health Technology and Clinical Epidemiology, National Institute of Health Research and Development (NIHRD), Ministry of Health, Jakarta, Indonesia; Indonesia Research Partnership on Infectious Disease (INA-RESPOND), Jakarta, Indonesia; Australia National University, Canberra, Australia; Faculty of Medicine, Universitas Gadjah Mada/Dr Sardjito Hospital, Yogyakarta, Indonesia; Faculty of Medicine, Universitas Padjadjaran/Hasan Sadikin Hospital, Bandung, Indonesia; Sulianti Saroso, Infectious Disease Hospital, Jakarta, Indonesia; Faculty of Medicine, Universitas Diponegoro/Dr Kariadi Hospital, Semarang, Indonesia; Faculty of Medicine, Universitas Hasanudin/Dr Wahidin Sudirohusodo Hospital, Makassar, Indonesia; Faculty of Medicine, Universitas Indonesia/Dr Cipto Mangunkusumo Hospital, Jakarta, Indonesia; Faculty of Medicine, Universitas Airlangga/Dr Soetomo Hospital, Surabaya, Indonesia; Faculty of Medicine, Universitas Udayana/Sanglah Hospital, Denpasar, Indonesia; US, National Institute of Allergy and Infectious Disease, Bethesda, USA

**Keywords:** Clinical, Disease, Indonesia, Infectious, Network, Research, Trial

## Abstract

Nationally representative observational and translational research is needed to address the public health challenges in Indonesia due to the geographic disparity, recently decentralized health system, and diverse infectious disease priorities. To accomplish this, the Indonesian Ministry of Health in collaboration with the US National Institute of Health has established INA-RESPOND (Indonesia Research Partnership on Infectious Disease) – a clinical research network comprising 9 referral hospitals, 7 medical faculties, and 2 research centres across Indonesia. The network provides a forum to conduct research at a national scale and to address scientific questions that would be difficult to address in smaller research settings. Further, it is currently conducting multi-centre research on the etiologies of fever, sepsis, and tuberculosis. There are opportunities to leverage existing network resources for other public health research needs. INA-RESPOND is an Indonesian-led network in a country with diverse population groups and public health needs which is poised to collaborate with researchers, universities, donors, and industry worldwide. This paper describes the network and its goals and values, as well as the management structure, process for collaboration, and future vision.

## Background

Historically, Indonesian collaborations on health research have resulted in landmark findings and impact on public health. Such collaborations include Indonesia’s contribution to the global eradication of smallpox through the development of the smallpox recognition card in 1968, which was adopted by the World Health Organization and distributed worldwide [1], a vitamin A supplementation study that commenced in Indonesia in the 1970s, which established the link between vitamin A deficiency and childhood morbidity and mortality and which resulted in intervention policies [2–5], and the hepatitis B vaccine field trial in 1987 that demonstrated effective integration of the vaccine into the Expanded Program on Immunization schedule to streamline vaccine delivery [6]. These examples illustrate Indonesia’s interest in active engagement and commitment to health research collaborations both domestically and internationally. Indonesia is also working on incorporating research into its national agenda, where health research is governed by several regulations. These include regulations about shipment of clinical specimens and biological materials, data sharing, intellectual property rights, health research as a part of the national health system, transfer of technology, research results and publication, and community involvement, as well as more recently the requirement to register clinical research.

Infectious disease research and surveillance is managed by the Ministry of Health (MoH), Ministry of Research and Technology, and Ministry of Education. Research and surveillance under the MoH are conducted by three government agencies: (1) the National Institute of Health Research and Development (NIHRD), (2) the Directorate General of Disease Control and Health Environment, and (3) the Directorate General of Medical Services. Research at the Ministry of Research and Technology is mostly conducted by the Eijkman Institute for Molecular Biology, and at the Ministry of Education by the medical and public health faculties.

However, given Indonesia’s diverse geography, variation in population health, relatively recent decentralized health system, and the varied infectious disease profile, a nationally cohesive effort is needed to successfully address major health and infectious disease-related issues. Such a national forum would have the added advantages of bringing together researchers and helping them network within and outside the country, as well as build internal capacity. Additionally, it would be an invaluable resource in the face of a future emerging infectious disease threat.

Recognizing this, NIHRD engaged in a partnership with the United States National Institutes of Allergy and Infectious Disease (NIAID) within the National Institutes of Health (NIH) to develop a network that could potentially become a leading scientific collaboration in the country to address questions of national and global impact and to enhance the capacity for implementation research [7]. Since the MoH budget for research is limited (44 million US dollars in 2014) [8] and the priority is not for conducting clinical research, this partnership is advantageous as it can attract additional research funding by providing potential collaborators a well-developed infrastructure and facilities.

This paper describes the establishment of the INA-RESPOND network, its key goals, challenges, and current and future research.

### Indonesia research partnership on infectious diseases (INA-RESPOND)

**Figure 1:**
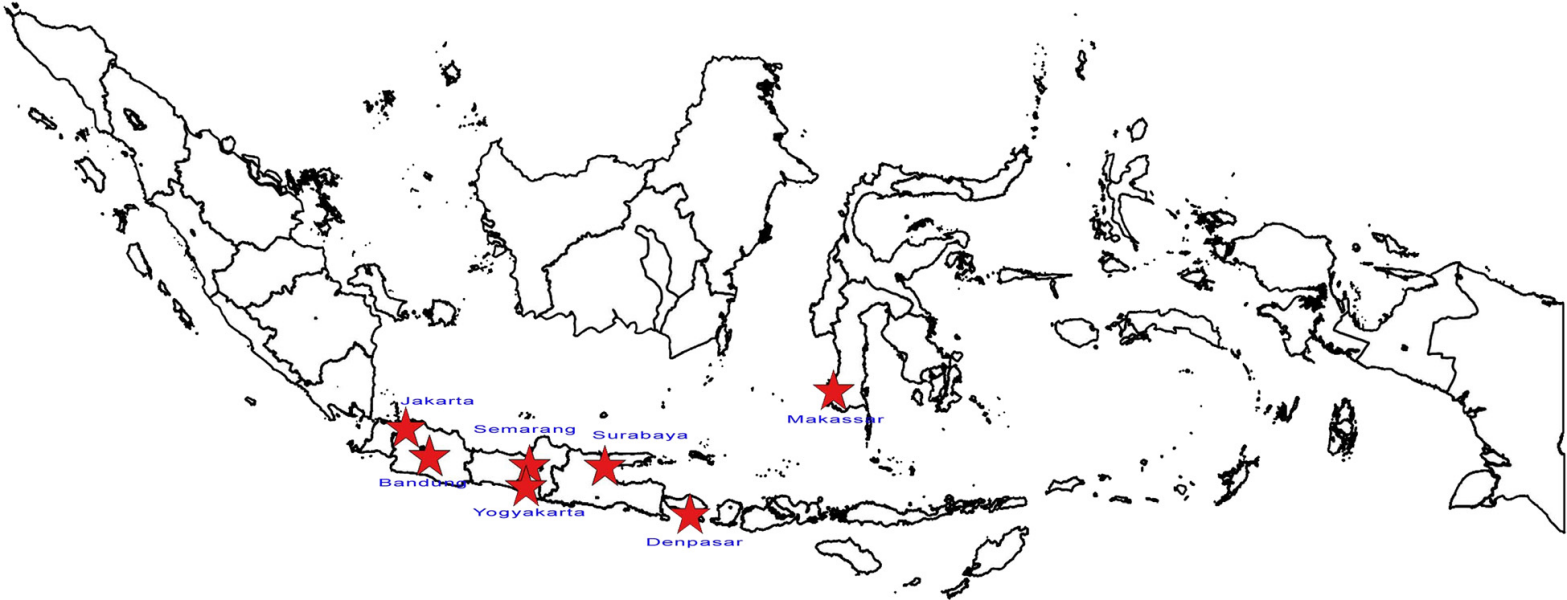
**The distribution of INA-RESPOND sites.**

Initial discussions to develop the network began in 2007 at the request of the Minister of Health of Indonesia at the time. In 2011, a Steering Committee was established to determine the initial framework and subsequently Ministerial and NIHRD decrees were issued to help with formalizing the structural integration of the network within the existing health framework of the country under the MoH. Presently, INA-RESPOND comprises 7 medical faculties and their 9 corresponding hospitals as well as the Eijkman Institute, located in 7 large cities on Java, Bali, and Sulawesi islands (Figure 1), under the coordination of NIHRD. The NIAID and the United States Centers for Disease Control and Prevention (US CDC) are collaborating partners within the network. The mission of the network is to build a sustainable and well-recognized research network that is able to conduct high-quality infectious disease research in order to improve the health of the people of Indonesia and to be beneficial to the international community. The network has a strategic plan and scientific agenda based on input from multiple stakeholders.

### Goals and values of the network

INA-RESPOND is committed to becoming a regional leader in clinical research excellence. The network is focused on three main goals. The first is to generate new knowledge on infectious diseases in the areas of pathogenesis, treatment, and prevention, and to ensure that relevant results are disseminated to public health officials so that there is real impact on policies. To accomplish this, the network has a scientific agenda focused on the national infectious disease health priorities as determined by the MoH. Secondly, there is a keen awareness that long term sustainability can only be achieved by establishing research infrastructure embedded within the current system, providing appropriate training and designating adequate human resources. INA-RESPOND supports a model of full time dedicated and well-trained research staff to assist busy investigators so that research can be integrated in busy clinical environments. Finally, the network hopes to develop, implement, and maintain strong internal and financial management and research operations and practices to ensure quality research can be supported by well-organized, coordinated, and comprehensive mechanisms.

The network is committed to conducting innovative, scientifically-sound, excellent, and ethical research, and is responsive to the health priorities in Indonesia. INA-RESPOND members work on the principles of teamwork based on trust, respect, transparency, good communication, collaboration, and shared responsibility. Anticipating centrality towards the most established members, which is commonly found [7], INA-RESPOND is enhancing the research and laboratory capacities in all the sites in order for sites to take turns as the principal investigators, and sites are able to perform standard laboratory assays.

### Organizational structure

**Figure 2:**
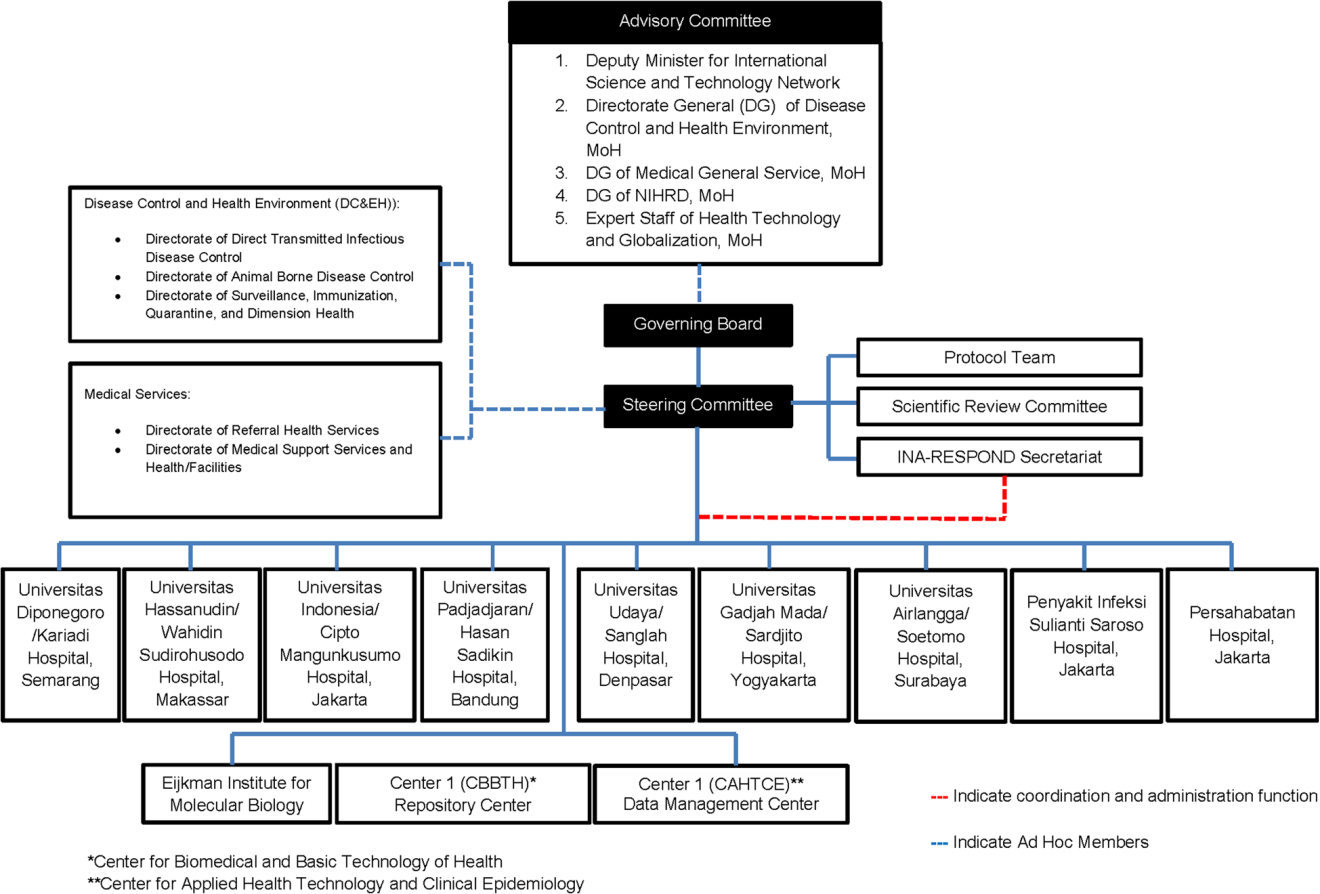
**The organizational structure of INA-RESPOND.**

To achieve a truly national research network, a comprehensive organizational structure was developed to include major stakeholders in the national research and public health community (Figure 2). The Advisory Committee is comprised of members from the Research and Technology Ministry, Directorate-General of NIHRD, Directorate-General of Disease Control and Environmental Health, Directorate-General of General Medical Services, and MoH expert on Health Technology and Globalization. This committee provides guidance and input on the development and support for the implementation of scientific activities within INA-RESPOND and ensures concurrence with national priorities.

The Governing Board is currently comprised of members from the two agencies engaged in a government-to-government collaboration, the Indonesian NIHRD, and the US NIAID, as well as the Chair and Vice Chair of the Steering Committee. It is tasked with ensuring that national health priorities are translated into the research agenda and are in line with Advisory Committee suggestions. It is also instrumental in identifying funding sources for the network and approving additional partners. The Steering Committee of the network has representatives of each participating hospital and institution, US NIAID, and CDC, and manages a day to day governance of the network, planning network activities, development of research protocols, approval of research projects, reviewing progress of the network, and identifying new partners.

Financial and technical support for the network comes from the NIHRD under the MoH and the US NIAID under the Department of Health and Human Services. Additionally, all partners contribute resources at their sites so that research is integrated within the hospitals. Unlike other collaborations or networks, which are often donor driven [7], all the strategic decisions are made by the Steering Committee and Governing Board, in which the donors have equal vote with other members.

### Current capabilities and functions

The INA-RESPOND secretariat is located at the NIHRD and directed by the Chair of the Steering Committee. It is the central operations and coordinating centre of the network and provides all support needed to the sites and the investigators in conducting research protocols. The secretariat supports and assists investigators in the development of protocols and study documents and provides regulatory support, trainings and monitoring, data management and analysis, centralized specimen repository, and laboratory support. The independent scientific advisory committee reviews network protocols and provides suggestions to ensure they are scientifically sound. The INA-RESPOND data safety and monitoring board provides oversight to interventional clinical trials.

In keeping with the commitment that research capacity must be developed nationally, the sites are an integral part of this effort. The network has provided initial infrastructure, including laboratory information management system software, office and laboratory supplies, and freezers to store the specimens. Each site has Good Clinical Practice and Good Laboratory Practice trained staff dedicated to research and fully engaged in research activities.

### Research implementation and current studies

As INA-RESPOND aims to answer questions based on national health priorities, studies are conducted in several sites to ascertain adequate representation of the diverse demographical population. The network hopes to expand in the future to include additional regions of the country.

Research priorities identified and proposals received are evaluated by the Steering Committee based on country priorities as outlined by the MoH, potential impact, capacity building elements, current capabilities, and resource allocation expected from the network. Selected ideas are forwarded to the Governing Board for development prior to protocol. INA-RESPOND is preparing guidelines that describe these procedures for potential collaborators as well as network members.

Protocols are reviewed by a scientific review committee at the NIHRD, followed by the Research Ethics Committee/Institutional Review Board of record. To facilitate regulatory approvals, INA-RESPOND has developed reliance agreements between multiple Institutional Review Boards to circumvent the need for multiple approvals.

The first study of the network was developed with two clear goals: to understand the etiologies of fever requiring hospitalization so that areas of future research could be identified and to establish clinical research infrastructure. The second study is collaboration with the South East Asia Infectious Disease Clinical Research Network to determine the etiologies, management, and outcomes of sepsis and severe sepsis. Subsequent studies that are being developed are directly based on several key priority areas of the MoH, which are tuberculosis and HIV. The tuberculosis study aims to estimate the proportion of multi-drug resistant tuberculosis in naïve and previously treated patients, treatment outcomes, and factors associated with these outcomes. The HIV cohort study aims to collect epidemiological, clinical, and laboratory data from HIV patients throughout Indonesia using a nationally standardized form.

### Challenges

INA-RESPOND presented a unique approach to research in Indonesia. As a first step, it was very important to engage stakeholders, researchers, national health programs, and policymakers. Given that the more common paradigm was of externally sponsored research, it was important to encourage clinicians and health policymakers to identify research priorities in Indonesia and enhance the understanding that the sustainability of the network depended on their own active and continued participation. Engaging busy clinicians in all research processes, from the preparation of study protocols to publication, and recruiting full-time research assistants at the sites that were interested in a research career were also priorities.

To respond to these, INA-RESPOND engaged in meetings with stakeholders and developed an Advisory Committee (as described above). On an ongoing basis, the network holds regular trainings, workshops, and seminars to build trust by involving and regularly updating all parties on the network activities. The network also provides opportunities for members to travel to national and international research conferences in areas of interest, holds regular Steering Committee meetings, and issues a newsletter to review network progress. Researchers, stakeholders, and research assistants are engaged in development of research concepts and protocols. Future plans include supporting researchers to complete master and doctorate degrees.

### Future network vision and collaborations

Developing a network requires collaboration and partnership at many different levels. Key to success is developing trust within the research community in Indonesia and internationally. INA-RESPOND does not wish to be seen as a collaboration of a select group of scientists but wishes to be a support and resource for all investigators in Indonesia and globally. The network is committed to identifying and establishing new collaborations and partnerships that can enhance its mission. The eventual goal would be for researchers to be able to submit proposals to the network and, if approved, the network would provide support in part or complete so that investigators can leverage network resources rather than investing in these individually. It would also enable investigators to envision research questions that could be of much larger national significance and on a much larger scale than could be accomplished by a single hospital or centre.

The network aims to establish clinical research facilities across the country to enable readiness in the event of emerging infectious disease transmission. Once research facilities exist they can be leveraged for non-infectious disease research as well. While research on infectious diseases is the current research focus of the network, INA-RESPOND is also interested in collaborating in non-infectious and chronic diseases since the threat of these is also emerging [9]. A soon to commence study will include a collaboration with the US National Cancer Institute of the National Institutes of Health to evaluate smoking as a risk factor in diseases.

The establishment of a specimen bio-repository is also a valuable asset for the country. These specimens may be utilized in the future to conduct research based on new findings or emerging research needs.

The network believes that, to be true to its goals of building research capacity, it must provide resources for new and young investigators, such as trainings in manuscript and grant writing, ethics, and eventually Masters and PhD programs, so that a constant infusion of new and well trained investigators can be maintained.

## Conclusion

INA-RESPOND is Indonesia’s first clinical research network supported by the MoH. The aim is to conduct translational and clinical research and to prevent and treat infectious diseases based on national concerns and in alignment with MoH priorities. It is intended that the research conducted by this network will support the development of public health policies and build sustainable research capacity within Indonesia. Multiple funding streams will be key to ensuring financial strength and sustainability. Future plans include expansion to additional sites so that optimal geographical and demographical representation can be achieved and engagement with new collaborators, including industry and other sponsors, in order to achieve the network mission of improving the health of the people of Indonesia and the international community.

## Abbreviations

INA-RESPOND: Indonesia research partnership on infectious diseases
MoH: Ministry of Health
US-NIAID: United States National Institutes of Allergy and Infectious Disease
NIHRD: National Institute of Health Research and Development
US CDC: United States Centers for Disease Control and Prevention
